# Effects of Digested Onion Extracts on Intestinal Gene Expression: An Interspecies Comparison Using Different Intestine Models

**DOI:** 10.1371/journal.pone.0160719

**Published:** 2016-09-15

**Authors:** Nicole J. W. de Wit, Marcel Hulst, Coen Govers, Jan van der Meulen, Angeline van Hoef, Geert Stoopen, Astrid Hamers, Arjan Hoekman, Ric de Vos, Toine F. H. Bovee, Mari Smits, Jurriaan J. Mes, Peter J. M. Hendriksen

**Affiliations:** 1 Food & Biobased Research, Wageningen University and Research Centre, Wageningen, The Netherlands; 2 Wageningen Livestock Research, Wageningen University and Research Centre, Wageningen, The Netherlands; 3 RIKILT-Institute of Food Safety Wageningen University and Research Centre, Wageningen, The Netherlands; 4 Plant Research International, Wageningen University and Research Centre, Wageningen, The Netherlands; Ludwig-Maximilians-Universitat Munchen, GERMANY

## Abstract

Human intestinal tissue samples are barely accessible to study potential health benefits of nutritional compounds. Numbers of animals used in animal trials, however, need to be minimalized. Therefore, we explored the applicability of *in vitro* (human Caco-2 cells) and *ex vivo* intestine models (rat precision cut intestine slices and the pig *in-situ* small intestinal segment perfusion (SISP) technique) to study the effect of food compounds. *In vitro* digested yellow (YOd) and white onion extracts (WOd) were used as model food compounds and transcriptomics was applied to obtain more insight into which extent mode of actions depend on the model. The three intestine models shared 9,140 genes which were used to compare the responses to digested onions between the models. Unsupervised clustering analysis showed that genes up- or down-regulated by WOd in human Caco-2 cells and rat intestine slices were similarly regulated by YOd, indicating comparable modes of action for the two onion species. Highly variable responses to onion were found in the pig SISP model. By focussing only on genes with significant differential expression, in combination with a fold change > 1.5, 15 genes showed similar onion-induced expression in human Caco-2 cells and rat intestine slices and 2 overlapping genes were found between the human Caco-2 and pig SISP model. Pathway analyses revealed that mainly processes related to oxidative stress, and especially the Keap1-Nrf2 pathway, were affected by onions in all three models. Our data fit with previous *in vivo* studies showing that the beneficial effects of onions are mostly linked to their antioxidant properties. Taken together, our data indicate that each of the *in vitro* and *ex vivo* intestine models used in this study, taking into account their limitations, can be used to determine modes of action of nutritional compounds and can thereby reduce the number of animals used in conventional nutritional intervention studies.

## Introduction

Animal experiments for human purposes are only acceptable if they are expected to yield a proven benefit for humans. For research on drug development and human diseases this benefit is clear. However, for testing potential health improving effects of nutritional additives moral justification is more controversial. On the other hand, epidemiological data alone are not sufficient to sustain claims on health effects [1]. Therefore, when indications for health improving effects are obtained, it is important to detect the mechanism responsible for this effect. Transcriptomics analysis has been demonstrated to be a valuable method for detecting modes of action [2,3,4]. An important issue, hereby, is whether *in vitro* or *ex vivo* models provide sufficient information or that *in vivo* experiments remain essential.

To investigate this issue, the present study assessed the performance of three intestinal models that either used no animals (human Caco-2 cells) or a limited number of animals (*Ex-vivo* rat precision cut intestine slices, and the pig *in-situ* small intestinal segment perfusion (SISP) technique). Each model system has its own strengths and weaknesses. The human Caco-2 cells that are originally of colonic origin can be differentiated into a small intestinal phenotype, which is frequently used as an *in vitro* model for absorption, bioavailability and subsequent (metabolic) responses to food compounds [5]. Caco-2 cell cultures can be tightly controlled and gene expression profiles in (post-confluent) Caco-2 cells reflect normal differentiated villus cells [6]. This makes Caco-2 cells a reliable model for detecting responses to food. However, it has to be kept in mind that Caco-2 cells are immortalized cells and consist of only one cell type, namely enterocytes. This might limit the translation of results obtained in Caco-2 cells to processes taking place in intestinal tissue with various cell types. Rat small intestine slices are composed of multiple cell types, making it more comparable to the *in vivo* situation. Moreover, multiple rat small intestine slices can be obtained per rat, enabling to study the response to food compounds by using a limited number of rats. After preparation, however, the rat slices have a limited lifespan. Exposure experiments have to be performed within a relatively short timeframe to avoid influences of tissue degeneration. In the pig SISP model, multiple loops of the small intestine are being used per pig, thereby reducing the number of animals needed per trial. The SISP model is closest to the *in vivo* situation. It is a multicellular system with intact blood flow. Immune cells, proteins and chemicals from the periphery can infiltrate the lamina propia of the intestinal loops and may activate feed-back mechanisms or opposite reactions in cells of the intestine [7]. The genetic variation between pigs can substantially influence the responses. An important goal of this study was therefore to investigate to which extent the modes of action derived from transcriptomics studies depend on the model.

To compare the three model systems and simulate a more real life situation, *in vitro* digested white and yellow onion extracts were selected as model food source (rather than testing a single pure nutritional compound). Each of the intestine models were incubated with the same extracts. Onions were selected due to their indications for positive health effects, including cardiovascular benefits, support for bone and connective tissue, anti-inflammatory, anti-hyperglycemic, anti-asthmatic, anti-bacterial and anti-carcinogenic activity [8,9,10,11]. Onions are members of the Allium family and various species are found in a wide range across the world. The composition, concentration and biological activity of the different compounds in onions vary between different species [9]. Although it is difficult to assign the health benefits to a particular compound in onion, the high flavonoid levels and the sulfur-containing molecules are frequently reported to be the most likely candidates [9]. Beneficial health effects are often linked to the antioxidant properties of onions. Not only do they protect animal cells against cellular damage by scavenging free radicals, onions are also reported to initiate oxidative defence mechanisms in the cells, such as induction of phase II detoxification and antioxidant enzymes (e.g. Glutathione-S-transferases (GSTs) and heme oxygenase (HMOX1) [8,9,12]). Although health effects of onions have been frequently studied, their direct effect on whole-genome intestinal gene expression has hardly been explored. The present analysis focused on effects on intestinal gene expression induced by both a white and yellow onion variety to identify a more common onion effect instead of onion-type specific effects.

Since human intestinal samples are often barely accessible and numbers of animals used in animal trials need to be minimalized, the main goal of our study was to apply *in vitro* or *ex vivo* models to study the effect of food compounds, in this case onions, on intestinal gene expression in order to obtain more insight into their modes of action. We compared the performance of the three models and discuss their applicability for detecting modes of action.

## Materials and Methods

### Samples

White onions (WO) were derived from Mexico (size 50–70 mm) and yellow onions (YO) originated from France (size 65–90 mm). We included two different types of onions (white and yellow, derived from different locations, Mexico and France) since we were interested in common onion effects. This should prevent that breeding line specific effects would be detected.

### *In vitro* digestion

Representative wedges of onions were taken, mixed with an equal amount of 140 mM NaCl / 5 mM KCl and mashed with a hand blender. The *in vitro* digestion protocol was mainly based on the paper of Vreeburg et al. with some slight modifications and in line with the standardized protocol as proposed by Minekus et al. [13,14]. In more detail, 20 grams of sample was transferred into a 50 mL tube, the pH was adjusted to 2 with HCl and 0.667 mL of 40 g/L porcine pepsin in 0.1 M HCl was added. The samples were then incubated for 30 min at 37°C. Subsequently, 1 M NaHCO_3_ was added to raise the pH to at least 5.8, followed by 0.95 mL of 4 g/L porcine pancreatin in 0.1 M NaHCO_3_ and 0.5 mL of a mixture of sodium taurocholate and sodium glycodeoxycholate (176 mM of each) in 0.1 M NaHCO_3_. The pH of the sample was adjusted to 6.5 with 1 M NaHCO_3_, flushed with nitrogen and the sample was subsequently incubated for 30 min at 37°C. After this incubation the pH of the sample was adjusted to 7.5 with 1 M NaHCO_3_ and the weight of the sample was adjusted to 30 g with 140 mM NaCl / 5 mM KCl. Samples were centrifuged for 45 min at 3023 × g at 4°C. The supernatant was taken, flushed with nitrogen and stored at −80°C until further use. For preparing the *in vitro* digestion control, 140 mM NaCl / 5 mM KCl was used to replace onion wedges.

### *In vitro*: Human Caco-2 intestine model: exposure of transwell grown Caco-2 cells and the trans-epithelial electrical resistance (TEER) measurements

ATCC derived Caco-2 cells were cultured in Dulbecco’s modified Eagle’s medium (DMEM; Gibco-Invitrogen, Bleiswijk, The Netherlands) with 4.5 g/L glucose, 0.58 g/L glutamine, no pyruvate, supplemented with 10% heat inactivated FBS (Hyclone Perbio, Etten-Leur, The Netherlands) and used with passage numbers between 30 and 60. For transwell assays, 330,000 cells were grown on ThinCert transwells with 33.6 mm^2^ membranes and 0.4 μm pores in 24-well suspension culture plates. Cells were grown for 21 days at 5% CO_2_ and 37°C and apical (150 μl) and basolateral (700 μl) medium were replaced three times per week.

Digested white onion (WOd), digested yellow onion (YOd) and digestion control samples (saline digest, Sd) were diluted 1:2 in DMEM/FBS medium. The pH was adjusted with sodium hydroxide when necessary, using phenol red in DMEM as indicator. The cell medium was replaced one day before the exposure experiments. TEER was measured before sample addition using a MilliCell-ERS Ώ meter (Millipore, Molsheim, France). Medium was removed from the apical and basal compartments and diluted samples were added in to the apical compartment while fresh DMEM/FBS medium was added to the basal compartment. TEER was determined directly and 6 hr after addition of samples to check integrity of the intestinal monolayer and exclude toxic effects of the digested onion samples. In each experiment, the onion and control samples were exposed to Caco-2 cells in triplicate and three independent exposure experiments were performed at different days. After 6h incubation, the Caco-2 cells were lysed with 200 μL TRIzol (Invitrogen, Life Technologies, Bleiswijk, Netherlands) and the triplicates in each experiment were pooled for RNA isolation.

### *Ex vivo*: Rat precision cut intestine slices: preparation, exposure and ATP measurements

The procedure for preparing rat precision cut slices has been extensively described by [15] and [16]. Approximately 8 weeks old male Sprague-Dawley rats were sacrificed and the small intestine was taken out as quickly as possible and directly put in ice cold Krebs-Henseleit buffer (KHB) previously flushed with carbogen gas (5% CO2 and 95% oxygen). Adhering fatty tissue was removed and the jejunum was cut in segments of approximately 3 cm which were flushed with ice cold oxygenated KHB. The segments were tightened at one end with surgical thread and filled with 3% agarose in 0.9% NaCl of 37°C until the intestine segments reached their normal diameter of approximately 5 mm. The segments were closed using forceps and immediately transferred into ice cold oxygenated KHB, turning the agarose into a flexible gel. Segments were cut into two halves with a surgical blade and each segment half was transferred to a precooled cylindrical mold-plunger assembly of the tissue-embedding unit (Krumdieck, Alabama Research and Development, Munford, AL). The mold was filled with 3% agarose of 37°C which was allowed to solidify into a gel. The plunger was removed and the jejunum embedded in agarose was placed in a Krumdieck slicer previously conditioned with ice cold oxygenated KHB. Slices of ~335 μm thickness were cut and collected in a beaker with ice cold oxygenated KHB. The slices were transferred into a beaker containing Williams’ Medium E of 37°C (WME) supplemented with glucose (25 mM), gentamicin (50 μg/mL), amphotericin B (2.5 μg/mL) and 5% FCS. Three slices were added to each well of 6-well plates that contained 2.0 mL supplemented WME and 0.4 mL sample extract (6 times diluted sample extracts). These 6-well plates were pre-incubated for at least 1 h at 5% CO_2_, 80% O_2_ at 37°C. The plates were immediately placed back in the oxygenated incubator equipped with a reciprocal shaker and incubated for 6 h at 5% CO_2_, 80% O_2_, 37°C and 35 cycles per min. Each sample was tested in six-fold while the non-digested 140 mmol L^−1^ NaCl / 5 mmol L^−1^ KCl control (physiologic salt control = Sal) was tested in twelve-fold. After exposure, the individual intestinal slices were dried by slightly touching a piece of Whatman paper, frozen in liquid nitrogen and stored at -80°C until mRNA isolation and ATP measurements.

For ATP measurements, 2 mL tubes (Simport, screw cap, Beloeil, Canada) were previously filled with 10 beads (Biospec Products, Zirconia Silica Beads, d = 2.3 mm, Bartlesville, OK) and 400 μL CelLytic buffer (CelLytic MT Cell Lysis Reagent, Sigma-Aldrich, Zwijndrecht, the Netherlands) and placed on ice. The frozen slices were transferred to these tubes, shortly vortexed and placed in a homogenizer (Bertin Technologies) for 2 times 15 s at 6500 x g. The tubes were centrifuged in an Eppendorf centrifuge at maximal speed and 100 μL supernatant was added to a tube containing 50 μL ATP-cell lysis solution. 50 μl of this solution was transferred to a well of a 96-well plate that already contained 100 μl of PBS. The plate was briefly mixed and 50 μl of substrate solution (Perkin Elmer, Oosterhout, the Netherlands) was added to each well. Luminescence at 590 nm was quantified as readouts by using a plate reader (SynergyTM HT Multi-Detection Microplate Reader, BioTek instruments, Winooski, VT). For the microarray experiment, exposures were done at the same day using two rats. This study was performed according to the national guidelines for the care and use of laboratory animals after approval of the animal welfare committee of Wageningen University, code 2014078.

### *In situ*: Piglets intestine and net fluid absorption using the SISP model

The *in situ* piglet model was used in combination with the small intestinal segment perfusion (SISP) technique as described before [17,18]. In this *in situ* test, eight 5 week-old male piglets (8.2 kg) were tranquillised with azaperone and anaesthesia was induced and maintained with sevoflurane and nitrous oxide. The piglets were placed in dorsal recumbence and an incision was made in the abdomen lateral to the linea alba. The abdominal cavity was opened and a first small intestinal segment was prepared by placing a thin cranial tube (inflow) and a wide tube (outflow) about 20 cm distal to the first. A second segment was prepared in the same way adjacent to the first and these two segments formed a pair. Within each piglet five paired segments were prepared at 31–58% along the total length of the small intestine; each segment with intact blood supply and innervation. At t = 0 segments were filled with 20 ml of Saline control solution (9 g NaCl, 1 g Bacto casaminoacids (Difco), and 1 g glucose/l distilled water), 20 ml onion digest (YOd or WOd) diluted 1:1 in peptone, or 20 ml control digest (Sd) diluted 1:3 in Saline solution. After 30 min, perfusion (2 ml/15 min) was started simultaneously for all segments, manually with syringes attached to the cranial tubes for a period of 6 h, with the non-absorbed fluid draining freely out of the distal tube into a corresponding drainage bottle placed at the same level as the piglet’s abdomen. Perfusion was stopped and for preparation of mucosal scrapings 10 cm of the caudal part of each segment was dissected. The mucosal samples were scraped, so mainly epithelial and mucosal cells were collected (approximately 1 g/sample). These samples were frozen in liquid nitrogen and stored at -80°C. In the first 4 piglets (number 1–4), YOd and Sd samples were distributed over a pair of segments, and over the piglets according to a Latin-square design. In the second 4 piglets (number 5–8) WOd and Sd samples were distributed over segments and piglets in the same manner as in piglets 1–4. Piglets were tranquillised with azaperone and anaesthesia was induced and maintained with sevoflurane and nitrous oxide. Piglets were euthanized with an overdose sodium pentobarbital”. The study was performed according to the national guidelines for the care and use of laboratory animals after approval of the animal welfare committee of Wageningen University, code 2012076.

### RNA isolation

For Caco-2 cells, cell lysates of triplicates per experiment were pooled and total RNA was extracted using the QIAshredder and RNeasy Mini kits (Qiagen, Venlo, The Netherlands) as described previously according to the manufacturers’ protocols [13]. Briefly, TriZol (Invitrogen) extraction from ThinCerts transwells was performed with 200 μL TriZol and followed by DNase-I treatment (Sigma-Aldrich, Zwijndrecht, the Netherlands) and RNeasy clean-up (Qiagen, Venlo, The Netherlands). Quality and amount of RNA was evaluated by UV spectrometry (260 and 280 nm wavelength) on the Nanodrop spectrophotometer (Thermo Scientific, Wilmington, DE, USA).

For the rat intestine, the mRNA was extracted using the QIAshredder and RNeasy Mini kits (Qiagen, Venlo, The Netherlands) according to the manufacturers’ protocols with minor modifications. In short, the frozen slices were transferred to tubes containing 10 beads and 600 μL RLT buffer with 1% β-mercaptoethanol, shortly vortexed and placed in a homogenizer (Bertin Technologies) for 2 times 15 s at 6500 x g. The samples were centrifuged in an Eppendorf centrifuge at maximal speed and the supernatant was transferred to a clean tube. From this point, the procedure continues according the manufacturer’s protocol and the quality and amount of RNA was evaluated by UV spectrometry on a Nanodrop spectrophotometer.

For the pig SISP segments, total RNA was isolated from mucosal scrapings with TRIzol^®^ reagent (Invitrogen) and treated with DNase as described [19]. The RNA was further purified using QIAamp MinElute Virus Spin Kit columns (Qiagen, Venlo, the Netherlands) according to the manufacturers’ instructions. The quality and integrity of the RNA samples were analyzed using Agilent Lab-on-a-Chip and Bioanalyzer (Agilent Technologies, Amstelveen, The Netherlands). All samples scored a RNA integrity number (RIN value) of ≥ 9.

### Gene expression analysis

RNAs of each independent Caco-2 or rat intestine experiment (n = 3 per treatment) were hybridized to Affymetrix Human Gene 1.1 ST and Rat 1.1 ST arrays according to standard Affymetrix protocols. Quality control of the datasets was performed using Bioconductor packages [20,21] integrated in an on-line pipeline [21]. Array data were normalized using the Robust Multiarray Average (RMA) M-estimator method [22,23], probe sets were defined according to Dai et al. [24]. To exclude interference of non- or very lowly expressed genes, genes were floored to an intensity by which 40% of the lowly expressed genes obtained this flooring value. To identify differential gene expression induced by WOd and YOd, paired-wise comparison analyses were performed (WOd/Sd and YOd/Sd) and genes with a LIMMA raw p-value <0.05 and fold change (FC) >1.5 or <-1.5 were selected for further data analyses.

Regarding the porcine microarray analysis, custom prepared 8x60K Agilent pig arrays G2519F Sus scrofa (035953; V2026440) containing 43,803 probes were used for single dye hybridizations with Cy3 labelled cRNA. Labelling, hybridization, scanning and feature extraction were performed in the same manner as described recently with minor differences [25]. Briefly, 500 ng RNA of each sample was amplified and labelled with the One-Color Microarray-Based Gene Expression Analysis Low input Quick Amp Labelling kit and 600 ng of Cy3 labelled cRNA was used for hybridisation on each patch. Hybridisation and washing of the arrays was performed according to the protocol described in the manual of the kit used for labelling of the RNA (Agilent Technologies). Arrays were scanned using a DNA microarray scanner with Surescan high resolution Technology (Agilent Technologies). Agilent Scan Control with resolution of 5 μ, 16 bits and PMT of 100%. Feature extraction was performed using protocol 10.7.3.1 (v10.7) for 1 colour gene expression. Gene expression in a small intestine segment was always compared to that of a segment of the same piglet (isogenic comparisons). Raw intensity values of probes were extracted from array data files created by the Agilent feature extraction protocol. Gene expression was floored using the same procedure as described above for the Affymetrix arrays. When multiple spots encode for the same gene, the spot with highest expression value was selected. Log2 normalised intensity values measured for YOd and Sd treated segments of piglet 1, 2, and 4 were loaded in the LIMMA software package to identify genes significantly differentially expressed with a p-value < 0.05 in YOd/Sd comparisons (paired statistical analysis). The same was done for the WOd/Sd comparisons in piglet 5, 6, 7, and 8. In addition to annotation provided by Agilent (pig) oligonucleotide sequences of differentially expressed probes not annotated yet, or not annotated in Unigene, tentative consensus sequences (TC) or mRNA accession number, were compared with the NCBI non-redundant nucleotide databases using ‘blastn’ to assign a gene-name to these probes. For 28369 probes on the pig array a significant match with a eukaryotic mRNA/gene could be assigned. Probes that did not produce a significant match were excluded from gene lists used for functional analysis. Throughout this manuscript official human gene-symbols (HUGO Gene Nomenclature Committee: http://www.genenames.org) were used in the text and in all (supplementary) figures and tables.

The microarray data are available at the Gene Expression Omnibus (GEO), http://www.ncbi.nlm.nih.gov/geo/: GSE83893 (human Caco2 cells), GSE84179 (rat intestine) and GSE83908 (pig SISP model).

### Pathway analysis

The Database for Annotation, Visualization and Integrated Discovery (DAVID version 6.7) website [26] and the “GeneAnalytics” expression-based analysis program (LifeMap Sciences, Inc. a subsidiary of BioTime, Inc., Alameda, CA) were used to assign genes to pathways. Since the annotation of human genes is more extended than that of rat and pig genes, the human gene annotation was used for this functional analysis. From DAVID, pathways (KEGG and Reactome) with an EASE score (p-value) of ≤0.1 (default EASE score) were retrieved. From GeneAnalytics output files pathways and associations with chemicals/compounds were retrieved with a high or medium score (p-value <0.05). Using the protein interaction tool of DAVID (UCSC_TFBS module), differentially expressed genes were enriched for specific transcription factor binding sites (TFBS). TFBS with an EASE score ≤0.1 were retrieved from DAVID.

## Results

### Viability measurements

In Caco-2 cells, 6 h exposure to digested onion extracts induced a small increase in TEER (5–10%) compared to Sd (data not shown). This indicated that onions did not disrupt the intestinal monolayer, on the contrary, onions seem to slightly support barrier integrity’.

For the rat intestine slices, 6 h culture induced a decrease of ATP level between 17 to 33% when compared to non-incubated intestine slices without a significant difference between treatment groups (data not shown).

### Gene expression analysis for each individual intestine model

**Fig 1:**
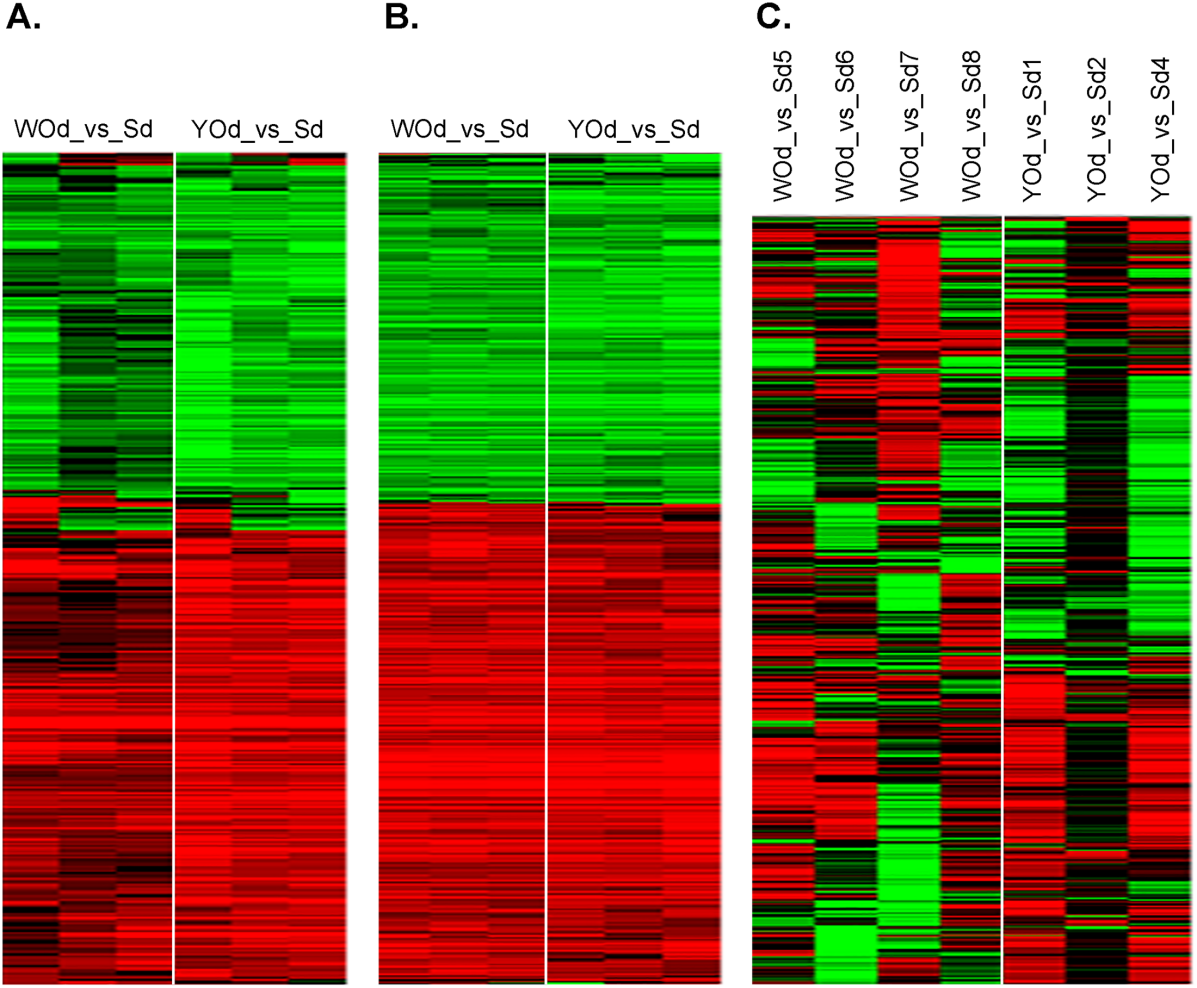
Unsupervised hierarchical clustering of genes altered by exposure to white onion or yellow onion digest. Genes were selected on > 1.5-fold up- or downregulation by WOd (WO_Dig) or YOd (YO_Dig) in at least 3 out of 6 (Caco-2 and rat intestine) or 7 (SISP) arrays. This resulted in 429, 766 and 713 genes for heatmaps of human Caco2 cells (A), rat intestine slices (B) and the porcine SISP model (C), respectively. Red: upregulation, green: downregulation, black: not affected. A ≥ 2-fold up- or downregulation obtained a maximal colour. The expression data for the three models (in 2log values of onion digest vs. saline digest) are provided are provided in S1, S2 and S3 Tables.

First we determined the effect of the two onion types in each of the three intestine models separately. For this, the effect of WOd and YOd on gene expression was compared to that of the digestion control samples (Sd). Both WOd and YOd clearly up- or downregulated groups of genes in Caco2 cells and rat intestine with relative little variation among the samples within the same exposure group (Fig 1A and 1B). The response to WOd and YOd was very much alike. In contrast, the response of the SISP model to the onion extracts was very variable, also between samples within the same treatment group and pig (Fig 1C).

**Fig 2:**
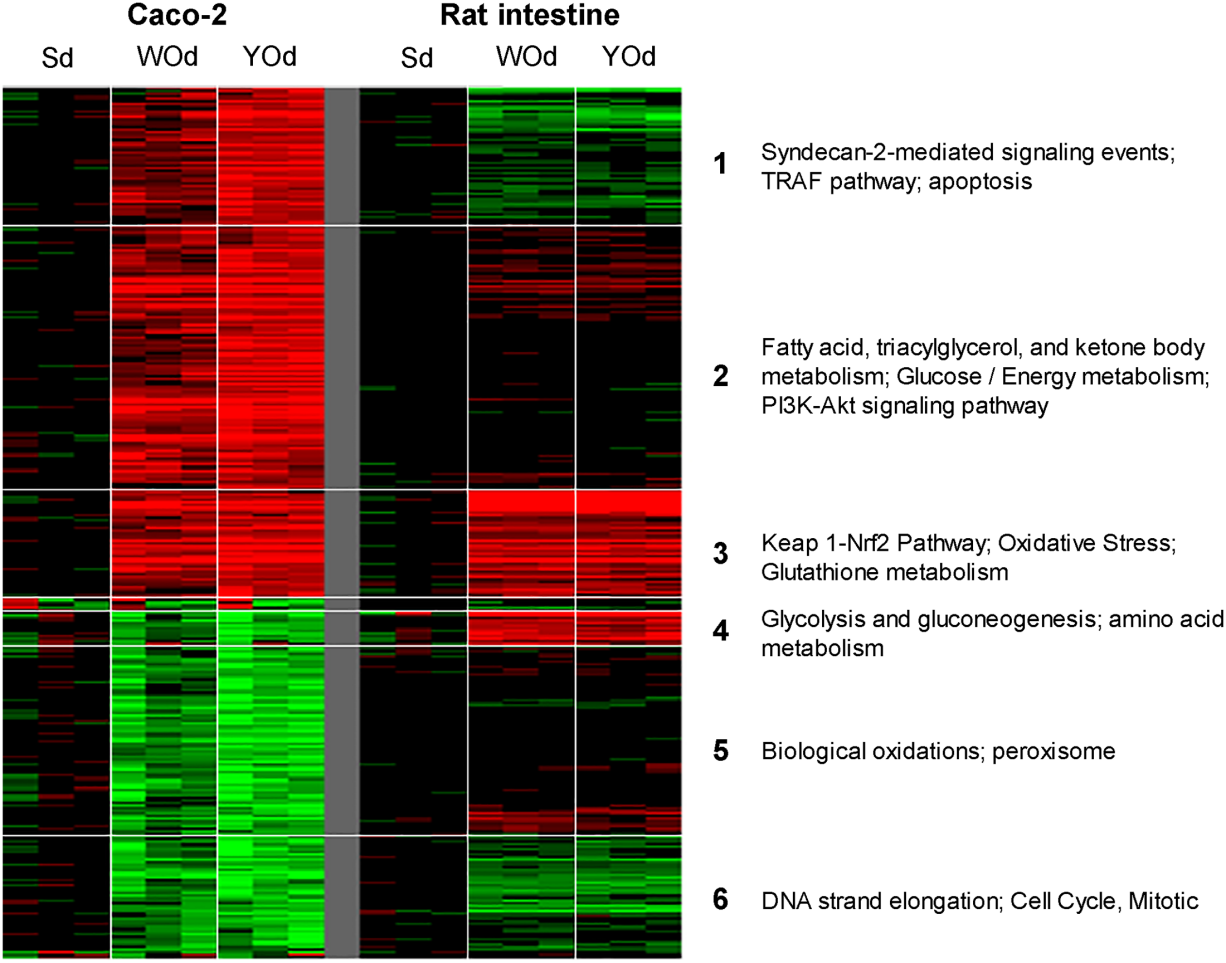
Comparison of the effects of digested white and yellow onion extracts on mRNA expression in human Caco-2 cells *in vitro* to that in rat intestine *ex vivo*. First, 429 genes were selected that were >1.5 fold up or downregulated in ≥3 of 9 arrays of the Caco-2 exposure as shown in Fig 1A. Thereafter, the mRNA expression data of the same genes for the rat intestine exposure were added, which was followed by unsupervised hierarchical clustering. WO_Dig or YO_Dig: White onion and yellow onion digested.

We then assessed to which extent genes that were up- or down-regulated in Caco-2 cells were also up- or down-regulated in rat intestine slices. Due to the high variation among the samples, the data of the SISP model were not included in this analysis. For this, 429 genes were selected that were >1.5-fold up- or downregulated in Caco-2 cells after which the expression data of the rat intestine slices were added. The expression data of these genes are provided in S4 Table. The clusters of genes were then analysed by pathway analysis. As shown in Fig 2, one group of genes (cluster 3) is upregulated both in Caco-2 cells and rat intestine slices. Genes in this cluster are involved in the oxidative stress response including the Keap1-Nrf2 pathway, and glutathione metabolism. Genes involved in the cell cycle were downregulated both in Caco-2 cells and the rat intestine (cluster 6). Genes involved in apoptosis related processes (cluster 1) and fatty acid and glucose metabolism (cluster 2) were mainly induced in Caco-2 cells whereas genes related to peroxisome were mainly downregulated in this cell line (cluster 5). Another group of genes (cluster 4) that are involved in glycolysis, gluconeogenesis and amino acid metabolism were downregulated in Caco-2 cells and upregulated in rat intestine slices. In Caco-2 cells but not in rat intestine slices, YOd induced stronger differential mRNA expression than WOd (Fig 2).

We continued the analysis by selecting genes that were represented at each of the three array platforms. While the human and rat arrays contained more than 19,000 genes, the pig array contained 13,587 annotated genes. The three types of microarrays shared 9,140 genes which were taken to compare the responses in number of significantly affected genes between the three models (Table 1). As a consequence, only genes affected in all of the three replicates were selected, i.e. changes in expression of genes present on one or two of the three types of microarrays were thus not included in further analysis. Differentially expressed genes with p-value < 0.05 (LIMMA raw/unadjusted p-value), in combination with a fold change (FC) > 1.5, were selected for further analyses. The highest number of genes was affected in the rat intestine, which was followed by Caco-2 cells. Few genes were affected in the pig model (Table 1). In the Caco-2 and pig SISP model, YOd had a more pronounced effect on gene expression than WOd. However, in general for each model, a high proportion of genes affected by WOd were also affected in the same direction by YOd. (Table 1, S2 Table).

**Table 1:** Differential gene expression per intestine model.

	# unique genes on array	genes present on each of type of array (proportions of all genes on array)	number of differentially expressed genes (FC>1.5, p<0.05^$^)		
			WOd/Sd	YOd/Sd	overlap WOd/Sd—YOd/Sd*
**Human Caco-2**	19715	9140 (46%)	111	372	93 (34↓, 59↑)
**Rat slices**	19312	9140 (47%)	377	395	265 (103↓,162↑)
**Pig SISP**	13587	9140 (67%)	7	27	4 (0↓, 4↑)

FC = fold change (numerical, either up or downregulated);

^$^ = LIMMA raw/unadjusted p-value.

* = ‘common onion effect’; affected in the same direction both by WOd and YOd.

WOd: white onion digest, YOd: yellow onion digest, Sd: control digest

### Overlap in onion-induced differential gene expression between the intestine models

**Fig 3:**
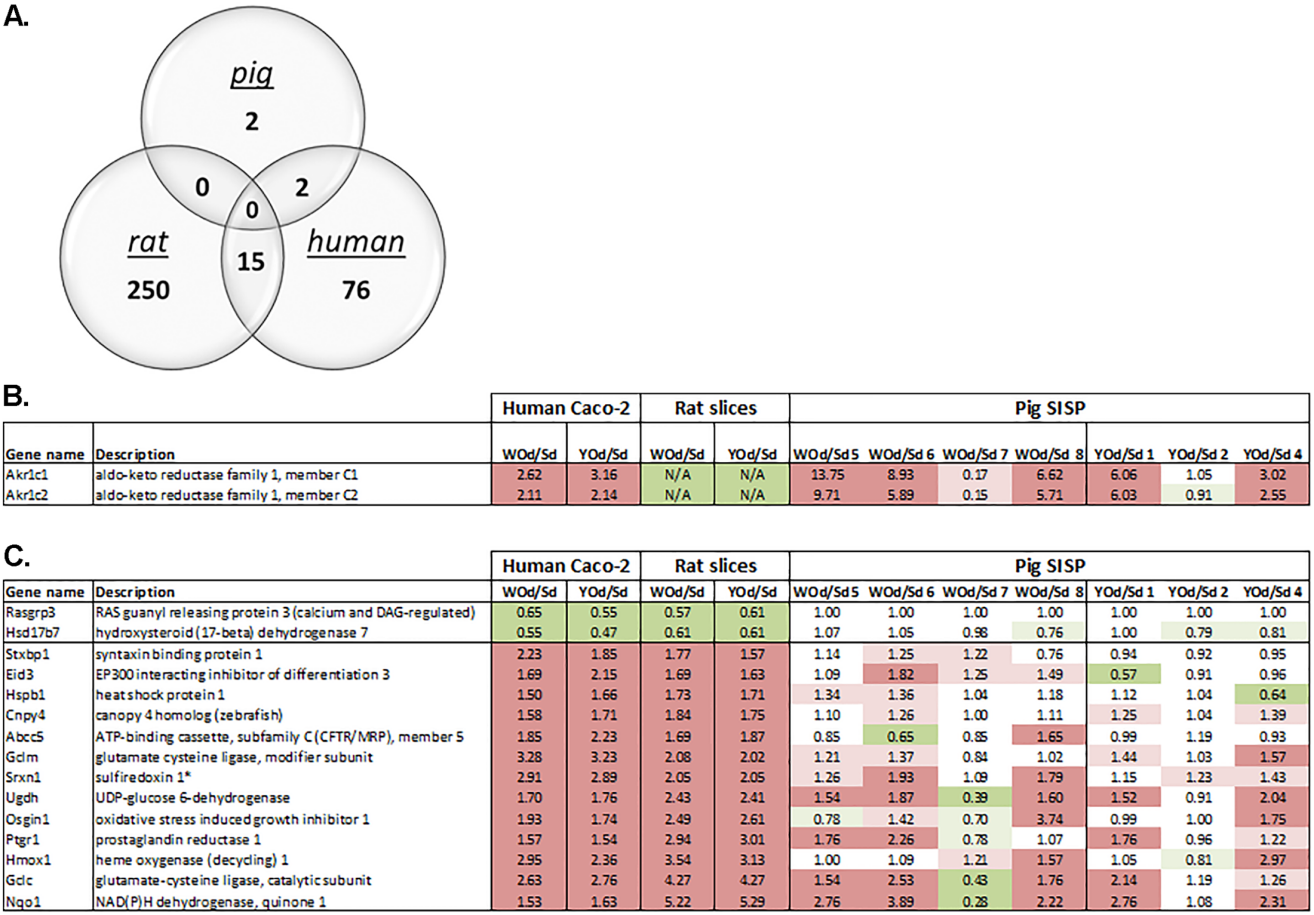
Overlap in onion-induced differentially expressed genes detected in human Caco-2 model, rat slices and pig SISP. A) Of 9,140 genes that were expressed in all three models, the overlap in onion-induced differential gene expression was determined (raw/unadjusted p-value<0.05 and FC > 1.5 or < 0.66). Expression of overlapping genes changed in the same direction (up- or downregulated). B) Overlap in differentially expressed genes (raw/unadjusted p-value<0.05) between human Caco-2 and pig SISP. C) Overlap in differentially expressed genes (raw/unadjusted p-value<0.05) between human Caco-2 and rat slices. Dark red and green indicates differential gene expression with fold changes >1.5 and <0.667, light red en green indicates fold changes >1.2 and <0.833. N/A; did not meet the 'flooring' criteria (low/no expression), so excluded from further analyses. * no homologues found in pig, only related genes (Sulfiredoxin-1-like).

To determine overlap in genes among the three intestine models in a more supervised manner, we focused only on genes that were significantly (p<0.05) affected with a minimal fold-change of 1.5 in the same direction by both YOd and WOd, (as indicated in the last column of Table 1). As shown in Fig 3A, Caco-2 cells and rat intestine had the highest overlap in genes (p-value <10^−7^, hypergeometrical distribution). The overlap in affected genes between the SISP model and Caco-2 was also significant (p-value < 10^−4^) but was based on only two genes, AKR1C1 and AKR1C2. None of the four genes affected by WOd and YOd in the SISP model were affected in the rat intestine slices. Based on the microarray data, AKR1C1 and AKR1C2 were not expressed in the rat intestine slices. The overlap between human Caco-2 and rat slices was most pronounced: fifteen genes were affected in the same direction in both rat intestine slices and human Caco-2: 13 were upregulated and 2 downregulated. Interestingly, Fig 3B and 3C show that the response to onion extracts of most of the pigs in the SISP model was quite comparable to that of the other two intestine models. Similar as observed in the heatmaps (Fig 1C), pigs 2 and 7 responded substantially different to the digested onion samples.

### Biological processes in the intestine affected by digested onions

**Fig 4:**
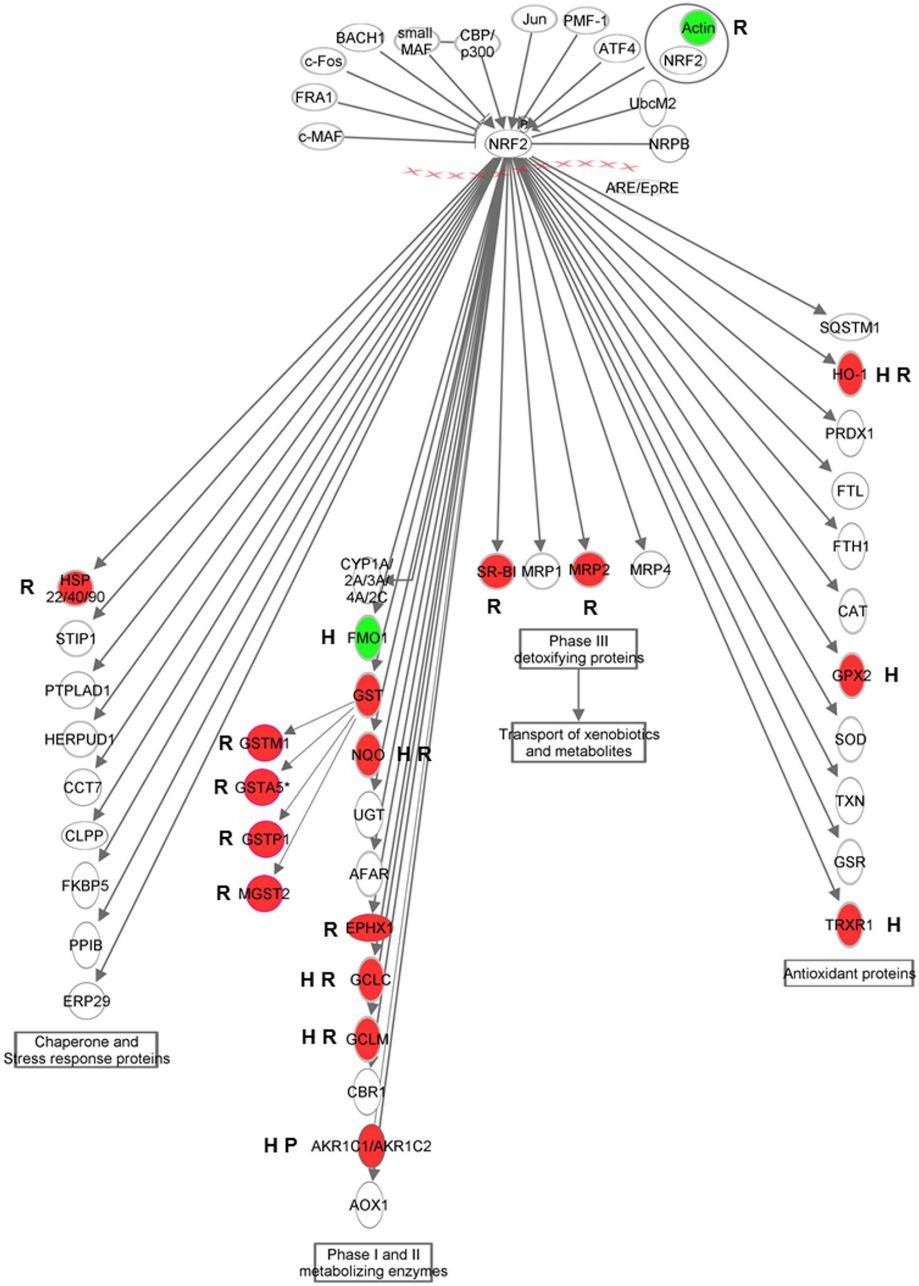
Onion-induced Keap1-Nrf2 pathway activation in human Caco-2 model, rat slices and pig SISP. Schematic visualisation of the Keap1-Nrf2 pathway, with upstream regulators and downstream target genes of Nrf2. Red indicates upregulation of the genes, green indicates downregulation. The letters next to the genes indicate the model(s) in which genes are affected: H: human Caco-2, R: rat slices, P: pig SISP.

Pathway analysis was performed for the set of (17) overlapping genes that were listed in Fig 3B and 3C (Table 2; upper panel) as well as separately for each set of genes affected in each of the individual models. Similar pathways were displayed separately (Table 2; middle panel) from pathways found exclusively for one of the models (Table 2; lower panel). Pathway analysis on the 15 genes affected both in human Caco-2 and rat intestine slices indicated activation of oxidative stress- and detoxification-related processes (Keap1-Nrf2 pathway, “oxidative stress”, “glutathione metabolism”) in these models and to some extent also in the pig SISP model. Within the Keap1-Nrf2 pathway, a relative high number of genes involved in phase II and III detoxification were induced in the rat intestine slices, whereas antioxidant proteins are mainly induced in human Caco-2 cells (Fig 4). Notably, the two AKR1C genes, members of the aldo/keto reductase superfamily that are induced by onion extracts in the pig SISP model, are also involved in the Keap-Nrf2 pathway (Fig 4). In agreement with results obtained with the 17 overlapping genes between at least two of the models (Table 2; upper panel), most pathways deduced from full lists of onion-affected genes (Table 2; middle panel) also showed substantial overlap in processes related to oxidative stress (e.g. oxidation-, detoxification- and glucuronidation-related processes), including involvement of AKR1C genes and transcriptional regulation by hypoxia induced factor 1 (HIF1), a process initiated when an overload of free radicals is sensed inside cells. Glucose/energy metabolism was also induced in both rat slices, pig SISP and human Caco-2 cells. On the other hand, immune-related pathways/processes, including helper T-cell pathways (Th1 and Th17), were significantly affected (induced or repressed) in rat slices and not in Caco-2 cells or the SISP model. Regulation of these processes indicates that cross-talk between enterocytes and resident immune cells occurred in the rat slices in response to onion extracts.

**Table 2:** Pathway analysis on onion-induced differentially expressed genes in human Caco-2, rat slices and pig SISP models.

Model(s)	process	pathway	# of genes	% match	GA score	DAVID score	genes^a^
		**Pathways affected in multiple models**					
Caco-2 and rat	oxidation	Keap1-Nrf2 Pathway	4	31	35	NA	GCLM, GGLC, HMOX1, NQO1
Caco-2 and rat	oxidation	Oxidative stress	3	9	21	NA	GGLC, HMOX1, NQO1
Caco-2 and rat	oxidation	Biological oxidations	4	2	18	NA	GCLM, GGLC, NQO1, UGDH
Caco-2 and rat	vitamin	One carbon pool by folate	3	4	18	NA	GCLM, GGLC, ABCC5
Caco-2 and rat	detoxification	Glutathione metabolism	2	4	12	NA	GCLM, GGLC
Caco-2 and pig	steroid	Synthesis of bile acids and bile salts	2	6	16	NA	AKR1C1, AKR1C2
Caco-2 and pig	steroid	Steroid hormone biosynthesis	2	4	15	NA	AKR1C1, AKR1C2
		**Pathways affected in individual models**					
Rat	vitamin	Vitamin A and carotenoid metabolism	5	12.5	15.3	NA	RBP7, BCO2, BCMO1, ADH4, SCARB1
Caco-2	vitamin	Vitamin A and carotenoid metabolism	3	7.5	12.0	NA	RARB, DHRS3, **ADH4**
Rat	vitamin	One carbon pool by folate	7	10.3	18.8	NA	SLC28A2, **PHGDH**, GCLM, GGLC, **MTHFS**, **MTHFD1L**, ABCC5
Caco-2	vitamin	One carbon pool by folate	3	4.4	9.8	NA	GCLM, GCLC, ABCC5
Rat	sugar/energy	Glucurodination	7	7.0	15.2	NA	CRYL1, AKR1B1, **GYS1**, PGM1, G6PC, UGDH, PYGM
Caco-2	sugar/energy	Glucurodination	5	5.0	16.2	NA	**HK2**, **GYS2**, **G6PC**, UGDH, **XDH**
Pig	sugar/energy	Glucose / Energy Metabolism	2	1.1	11.6	NA	PDX1, AKR1C2
Rat	sugar/energy	Glucose / Energy Metabolism	11	6.0	20.8	NA	**ARG1**, **SLC1A5**, **PDK1**, **BCAT1**, **PCK2**, **ACACA**, **GYS1**, **SCD**, IDH1, ABCC2, PPARGC1A
Caco-2	sugar/energy	Glucose / Energy Metabolism	5	2.7	12.2	NA	SLC7A11, HK2, CA2, AKR1C2, TXNRD1
Rat	sugar/energy	Galactose metabolism	5	13.9	16.0	NA	AKR1B1, **GYS1**, PGM1, G6PC, PYGM
Caco-2	sugar/energy	Galactose metabolism	3	8.3	12.5	NA	**HK2**, **GYS2**, **G6PC**
Pig	steroid	Synthesis of bile acids and bile salts	2	5.7	16.3	NA	AKR1C1, AKR1C2
Caco-2	steroid	Synthesis of bile acids and bile salts	3	8.6	12.6	NA	**ACOX2**, AKR1C1, AKR1C2
Pig	steroid	Steroid hormone biosynthesis	2	3.5	14.9	NA	AKR1C1, AKR1C2
Caco-2	steroid	Steroid hormone biosynthesis	3	5.3	10.5	NA	AKR1C1, AKR1C2, **HSD17B7**
Rat	oxidation	Oxidative stress	4	12.5	12.5	NA	GCLC, HMOX1, MAOA, NQO1
Caco-2	oxidation	Oxidative stress	5	15.6	24.2	NA	GCLC, HMOX1, TXNRD1, NQO1, **XDH**
Rat	oxidation	Keap1-Nrf2 Pathway	4	30.8	17.5	NA	GCLM, GCLC, HMOX1, **NQO1**
Caco-2	oxidation	Keap1-Nrf2 Pathway	4	30.8	23.5	NA	GCLM, GCLC, HMOX1, **NQO1**
Rat	oxidation	HIF1-alpha transciption factor network	5	7.7	12.0	NA	EGLN3, HMOX1, ADM, PGM1, **SMAD3**
Caco-2	oxidation	HIF1-alpha transciption factor network	3	4.6	10.0	NA	HMOX1, **HK2**, SERPINE1
Pig	oxidation	Biological oxidations	2	0.8	10.8	NA	AKR1C1, AKR1C2
Rat	oxidation	Biological oxidations (REACTOME_13433)	10	3.9	NA	0.002	GSTA4, GCLC, PTGS2, GSTA5, MAOA, ADH4, UGDH, GCLM, GSTP1, MGST2
Caco-2	oxidation	Biological oxidations	10	4.1	28.3	NA	GCLM, GCLC, **FMO1**, AKR1C1, AKR1C2, **ADH4**, **NAT8**, GPX2, NQO1, UGDH
Pig	detoxification	KEGG-Metabolism of xenobiotics by cytochrome P450	2	3.3	NA	0.023	AKR1C1, AKR1C2
Rat	detoxification	KEGG-Metabolism of xenobiotics by cytochrome P450	7	11.7	NA	0.002	GSTM2, GSTA4, GSTA5, ADH4, EPHX1, GSTP1, MGST2
Caco-2	detoxification	KEGG-Metabolism of xenobiotics by cytochrome P450	3	5.0	NA	0.095	AKR1C2, **ADH4**, AKR1C1
Rat	detoxification	Glutathione metabolism	8	14.0	24.6	NA	GCLM, GCLC, GSTA4, GSTA5, GSTP1, GSTM2, IDH1, MGST2
Caco-2	detoxification	Glutathione metabolism	3	5.3	10.5	NA	GCLM, GCLC, GPX2
Rat	ECM interactions	Integrin Pathway	17	3.2	18.1	NA	MAPK6, ITGB6, ITGA2, ITGA2B, ITGA6, ACTA1, CCL20, CCL5, CCL24, CXCL2, CXCL10, DOCK1, JAM3, MMP7, TGFBR1, LAMC2, LAMB3
Caco-2	ECM interactions	Integrin Pathway	7	1.3	9.9	NA	**GRB7**, CACNB3, WASF1, CLDN3, SERPINE1, GNAZ, **PIP5K1B**

^a^: genes in bold and underlined are downregulated; the non-marked genes are upregulated.

## Discussion

The present study assessed the performance of three intestinal models that either used no animals (cultured human Caco-2 cells), or a limited number of test animals by using *ex-vivo* rat precision cut intestine slices or the pig *in-situ* small intestinal segment perfusion (SISP) technique.

Both rats and pigs are often used as animal models for assessing digestibility of nitrogen and amino acids in humans [27]. The upper digestive tract (mouth to ileum) of rats is quite similar to that of humans both anatomically and physiologically [28]. For this reason, the growing rat has been recommended and is generally accepted as a valid animal model for predicting protein digestibility in humans [29].

The pig has been promoted as a useful model for human nutrition studies as well [30]. Except for primates, the pig is the laboratory animal nearest to humans in terms of anatomy and physiology. This is also true for the gastrointestinal tract, which has very close resemblance to human physiology, digestive function and splanchnic blood flow characteristics [27]. The protein digestibility in the ileum of the pig is quite similar to that of humans. Studies in which pigs and healthy adult human subjects were fed with semi-synthetic mixed meals that were 15N-labelled, containing either casein, hydrolysed casein or rapeseed isolate, showed that the true ileal N digestibility between the two species were quite equal. The largest difference was found for ileal N digestibility that was markedly lower (14–16%; P < 0·001) in human subjects than in pigs [27].

The human Caco-2 cells differ from the two models mentioned above since these represent a rather homogeneous cell population instead of a mixture of cell types.

Intestinal cells in all three models were exposed for 6 hours to a digested saline control and to digested extracts of white and yellow onions and subsequently analysed by transcriptomics. The main goal was to assess whether or not these models would indicate the same modes of action, or in other words, to which extent do modes of action depend on the cell model.

Since the SISP experiment resulted in variable results (Fig 1C), we first focused on a comparison between the human Caco-2 cells and the rat intestine slices by unsupervised clustering analysis (Fig 2). A first finding was that in both models, most of the genes affected by WOd were also affected in the same direction by YOd, indicating similar modes of action of the two onion species. In Caco-2 cells, but not in rat intestine slices, YOd induced higher mRNA induction or repression than WOd (Figs 1A and 2). Moreover, YOd exposure also resulted in a (slightly) higher number of differentially genes compared to WOd in all models, but especially in human Caco-2 cells (Table 1). Likely, Caco-2 cells seem to be more responsive to YOd than WOd, while these two onion species did not seem to differ in their main modes of action as is indicated by the heatmaps in Fig 2.

Secondly, unsupervised clustering analysis revealed that genes involved in the oxidative stress response, including the Keap1-Nrf2 pathway and glutathione metabolism are induced both in human Caco-2 cells and rat intestine slices (Fig 2, cluster 3). Pathway analyses including only the overlapping differentially expressed genes in Caco-2 cells and rat slices (Table 2; upper panel) also indicated that the Keap1-Nrf2 pathway is one of the most affected pathway induced by onions and also the AKR1C genes, upregulated in pig SISP and caco-2 cells are reported to be involved in this pathway [31]. Interestingly, elevated expression of Nrf2 target genes, as is seen in our study (Fig 4), is suggested to support stress resistance [32]. Also other oxidative stress-related pathways (e.g. oxidation, detoxification, glucuronidation [33]) were shown to be affected by onions in multiple intestinal models (Table 2; middle panel). Therefore, these oxidative stress-related responses seem to be a common, species- and model-independent onion-induced effect. This is of interest since beneficial health effects of onions are often linked to the antioxidant properties of onions [34,35]. Onions have been reported to protect animal cells against cellular damage by scavenging free radicals. Moreover, onions induce phase II detoxification and trigger expression of antioxidant enzymes (e.g. GSTs and HMOX1) to initiate oxidative defence mechanisms in the cells [8,9,12]. This is in accordance with our findings. The Nrf2/Keap1-mediated activation of phase II detoxifying enzymes, however, is not unique for onions but have been reported to be induced by other vegetables and plant extracts as well [36,37,12]. Recently, the antioxidant activity of onions was also validated in peripheral blood mononuclear cells of humans [38]. Although this study did not report on the effect on intestinal tissue, it indicates that also in humans onions can induce and antioxidant response.

Although we found that the Nrf2/Keap1 pathway was activated by onion extracts in all three intestinal models, the models slightly differed in the response of genes in this pathway (Fig 4). For instance in the rat model, cytosolic (GSTA4), ER-associated (MGST2) and secreted glutathione-S-transferases (GSTP1, GSTA5) were up-regulated, whereas these glutathione-S-transferases were not induced in the Caco-2 cells and SISP loops after 6 hours incubation with WOd and YOd (Fig 4). This differential activation might indicate that the onion-induced oxidative stress responses have a slightly different mechanism in the intestinal models or that the models react in a different time-frame.

A high variation in natural gene expression between individual SISP piglets was revealed by heatmaps of the treated SISP loops (Fig 1C). In a recent SISP study, we also observed unsynchronized and sometimes different responses in individual SISP piglets, even when a strong inflammatory response was induced by a challenge with Salmonella bacteria [19].

A response in the intestine to a natural feed product like onion is, most likely, quite mild. In this study, subtle differences in expression levels of genes induced by specific components within the onion extracts may have been overshadowed by the existing natural bias in expression levels of genes and may have not been recognized using the selection criteria we used in this study. Nevertheless, the two genes of which the mRNA expression was significantly affected by onion extracts are known to be involved in the oxidative stress response, which fits with the results of human Caco-2 and rat intestine slices.

Interestingly, in all three intestine models also glucose/energy metabolism related pathways are affected by exposure to onions (Fig 2, Table 2). This is in line with effects of onions on glucose metabolism in literature [11,39,40] that reported especially anti-hyperglycemic properties in animal and human studies.

Next to overlap in onion-induced modes of action within the three models, also model-specific pathways were detected (Table 2; lower panel). These differences in pathways activated in the three models can partly be explained by the characteristics of the models, for instance the single cell-type system of the Caco-2 model versus a multicellular (and even dynamic) model of the rat slices and pig SISP. Especially in the rat model more changes in gene expression were found that are linked to, for instance, immune responses and extracellular matrix. This seems to be logical as immune cells are not present in the Caco-2 model.

The main purpose of this study was to compare three intestinal models for their applicability to detect onion-induced modes of action. Although the number of significantly affected genes varied considerably between the three models, each of the models indicated oxidative stress response as the main pathway induced by onion exposure which agrees with findings in animal and human trials published before. Taking this into account, our data reveal that use of these models, despite their limitations, can indeed reduce the number of animals used in conventional nutritional intervention studies.

## Supporting Information

S1 Table Caco2_WOd_YOd_affected_genes. Effects of digested onion extracts on mRNA expression in human Caco-2 cells. A heatmap of these data is given in Fig 1A.
(XLSX)

S2 Table Rat_Intest_WOd_YOd_affected_genes. Effects of digested onion extracts on mRNA expression in rat intestine slices *ex vivo*. A heatmap of these data is given in Fig 1B.
(XLSX)

S3 Table Pig_Intest_WOd_YOd_affected_genes. Effects of digested onion extracts on mRNA expression in the pig SISP model. A heatmap of these data is given in Fig 1C.
(XLSX)

S4 Table Caco2_429 genes_linked_to 339 genes in the rat_Hierarchically clustered. Comparison of the effects of digested onion extracts on mRNA expression between Caco-2 cells *in vitro* and rat small intestine slices *ex vivo*. A heatmap of these data is given in Fig 2.
(XLSX)

S5 Table Significantly differential expressed genes were selected for each of the three models (Rat, Caco2 and Pig-SISP) on a p-value <0.05 and a Fold Change (FC; Yod or Wod over Sd) of < 1.5 (up) or < 0.66 (down). For SISP arrays, the FC of all individual test-pigs (denoted by a number) are presented. For all three species/arrays official human gene-symbols [Gene names] are provided according to the HUGO Gene Nomenclature Committee. The reader may sort specific lists of regulated genes by using the [up /down], [species-array], or ["array experiment" (e.g. Caco-2 Yod/Sd] headers.
(XLSX)
